# Global Molecular Epidemiology of Respiratory Syncytial Virus from the 2017−2018 INFORM-RSV Study

**DOI:** 10.1128/JCM.01828-20

**Published:** 2020-12-17

**Authors:** David E. Tabor, Fiona Fernandes, Annefleur C. Langedijk, Deidre Wilkins, Robert Jan Lebbink, Andrey Tovchigrechko, Alexey Ruzin, Leyla Kragten-Tabatabaie, Hong Jin, Mark T. Esser, Louis J. Bont, Michael E. Abram, the INFORM-RSV Study Group, Christina Naaktgeboren

**Affiliations:** (University Medical Center Utrecht, Utrecht University, Utrecht, The Netherlands);; (ReSViNET Foundation, Zeist, The Netherlands and King’s College London, London, United Kingdom);; (ReSViNET Foundation, Zeist, The Netherlands and University of Turku and Turku University Hospital, Turku, Finland);; (Pontificia Universidade Catolica de Rio Grande do Sul, Porto Alegre, Brazil);; (The University of Western Australia, Perth, Australia);; (Hospital Clínico Universitario de Santiago, Galicia, Spain);; (ReSViNET Foundation, Zeist, The Netherlands and University of the Witwatersrand, Johannesburg, South Africa);; (Fukushima Medical University School of Medicine, Fukushima, Japan);; (University Hospital Giessen and Marburg, Marburg, Germany);; (Université Paris XII, Créteil, France);; (McGill University Health Centre, Montreal, Canada);; (McMaster University, Hamilton, Canada).; a Microbial Sciences, BioPharmaceuticals R&D, AstraZeneca, Gaithersburg, Maryland, USA; b Clinical Pharmacology & Safety Sciences, BioPharmaceuticals R&D, AstraZeneca, San Francisco, California, USA; c Department of Paediatrics, Division of Paediatric Infectious Diseases, Wilhelmina Children’s Hospital, University Medical Centre Utrecht, Utrecht University, Utrecht, The Netherlands; d Department of Medical Microbiology, University Medical Center Utrecht, Utrecht, the Netherlands; e Data Sciences & Artificial Intelligence, BioPharmaceuticals R&D, AstraZeneca, Gaithersburg, Maryland, USA; f ReSViNET Foundation, Zeist, The Netherlands; g Julius Clinical, Zeist, The Netherlands; Cepheid

**Keywords:** evolution, genetic variation, molecular epidemiology, resistance, respiratory syncytial virus, surveillance

## Abstract

Respiratory syncytial virus (RSV) is the leading cause of lower respiratory tract infection among infants and young children, resulting in annual epidemics worldwide. INFORM-RSV is a multiyear clinical study designed to describe the global molecular epidemiology of RSV in children under 5 years of age by monitoring temporal and geographical evolution of current circulating RSV strains, F protein antigenic sites, and their relationships with clinical features of RSV disease. During the pilot season (2017–2018), 410 RSV G-F gene sequences were obtained from 476 RSV-positive nasal samples collected from 8 countries (United Kingdom, Spain, The Netherlands, Finland, Japan, Brazil, South Africa, and Australia). RSV B (all BA9 genotype) predominated over RSV A (all ON1 genotype) globally (69.0% versus 31.0%) and in all countries except South Africa. Geographic clustering patterns highlighted wide transmission and continued evolution with viral spread. Most RSV strains were from infants of <1 year of age (81.2%), males (56.3%), and patients hospitalized for >24 h (70.5%), with no differences in subtype distribution. Compared to 2013 reference sequences, variations at F protein antigenic sites were observed for both RSV A and B strains, with high-frequency polymorphisms at antigenic site Ø (I206M/Q209R) and site V (L172Q/S173L/K191R) in RSV B strains. The INFORM-RSV 2017–2018 pilot season establishes an important molecular baseline of RSV strain distribution and sequence variability with which to track the emergence of new strains and provide an early warning system of neutralization escape variants that may impact transmission or the effectiveness of vaccines and MAbs under development.

## Abstract (precis)

Respiratory syncytial virus (RSV) is the leading cause of lower respiratory tract infection among infants and young children, resulting in annual epidemics worldwide. INFORM-RSV is a multiyear clinical study designed to describe the global molecular epidemiology of RSV in children under 5 years of age by monitoring temporal and geographical evolution of current circulating RSV strains, F protein antigenic sites, and their relationships with clinical features of RSV disease. During the pilot season (2017–2018), 410 RSV G-F gene sequences were obtained from 476 RSV-positive nasal samples collected from 8 countries (United Kingdom, Spain, The Netherlands, Finland, Japan, Brazil, South Africa, and Australia).

## INTRODUCTION

Respiratory syncytial virus (RSV) is the leading cause of acute lower respiratory tract infection (LRTI) among infants and young children worldwide (1, 2). Most infections occur seasonally during the winter months in temperate regions, but with greater variability throughout the year in the tropics (3, 4). In 2015, RSV was associated with 33.1 million episodes of LRTI, 3.2 million RSV-related hospital admissions, and 118,000 deaths in children less than 5 years of age, predominantly in developing countries (2). Although prematurity and congenital lung or heart conditions are well-known risk factors for severe RSV LRTI, characterized by bronchiolitis and pneumonia, all children are at risk for RSV LRTI with primary RSV infection during infancy (2, 5).

Prevention of RSV LRTI in all infants is a major public health priority; however, despite many years of attempted vaccine development, there are no licensed vaccines (6). While palivizumab (Synagis) is the only approved passive monoclonal antibody approach for prophylaxis of RSV disease, it is recommended for use only with high-risk infants and children (7). Because there is no approved RSV prophylaxis for the broader population of healthy infants, more than 20 vaccine candidates and monoclonal antibodies (MAbs) are currently in clinical development (8). The most advanced candidate is nirsevimab—a potent, extended half-life MAb recently shown to significantly reduce medically attended RSV LRTI and hospitalization throughout the RSV season in healthy preterm infants in a phase 2b trial (9).

RSV is a nonsegmented, single-stranded, negative-sense RNA *Orthopneumovirus* belonging to the *Pneumoviridae* family (10). The attachment (G) and fusion (F) surface glycoproteins mediate viral entry and are both important antigenic targets for virus-neutralizing antibodies. Based on the genetic variability of the second hypervariable 2 region (HVR2) of the G gene, RSV strains are classified into subtype A or B and further characterized into different genotypes (11). In contrast, the F protein exhibits relative genetic and antigenic stability (12), making it a major target for vaccine and MAb development. The extracellular region of the mature F protein is a trimer of F_1_ and F_2_ subunits produced by cleavage of an inactive precursor F_0_ and exists in prefusion and postfusion conformations. Six antigenic sites (Ø and I to V) have been identified in prefusion and/or postfusion F proteins (13) with target epitopes for prophylactic neutralizing MAbs, including: palivizumab (site II), nirsevimab (site Ø), suptavumab (site V), and MK-1654 (site IV) (14).

As RSV immunization candidates reach the final stages of clinical development, the need for global monitoring of RSV molecular epidemiology becomes increasingly important to ensure their effectiveness during licensure and use. While prophylactic approaches invariably rely on conservation of neutralizing epitopes, RSV replication is inherently error-prone, resulting in natural polymorphisms (15). Selective immune pressure may further result in the emergence and spread of neutralization escape variants, allowing for immune and/or prophylaxis resistance. Finally, evolutionary dynamics of RSV genotypes may correlate with transmission between seasons (16) and disease severity among patient types (17).

The International Network For Optimal Resistance Monitoring of RSV (INFORM-RSV) study was established to describe global molecular epidemiology of RSV by monitoring temporal and geographical distribution of current circulating strains, with a focus on antigenic site changes that may confer selective advantages in transmission or resistance. Here, we describe geographic, demographic, and clinical distribution of RSV strains and sequence diversity of G genes and F proteins collected from mostly hospitalized infants in 8 countries across 4 continents during the pilot 2017–2018 RSV season.

## MATERIALS AND METHODS

### Study design.

INFORM-RSV is a prospective, multicenter, global molecular epidemiology study to investigate temporal and geographic diversity of RSV isolates collected from children less than 5 years of age who are admitted to the hospital or visiting the outpatient clinic and are not using preventive or treatment medication for RSV. Over the course of a 5-year period (2017–2022), 10 to 20 RSV-positive nasal samples will be collected per month per site each RSV season. Informed consent is obtained from parent(s)/legal representative(s) in accordance with the International Conference on Harmonization Guideline on Good Clinical Practice E6 (ICH-GCP) and applicable national and international regulatory requirements (18).

### Sample collection.

**FIG 1:**
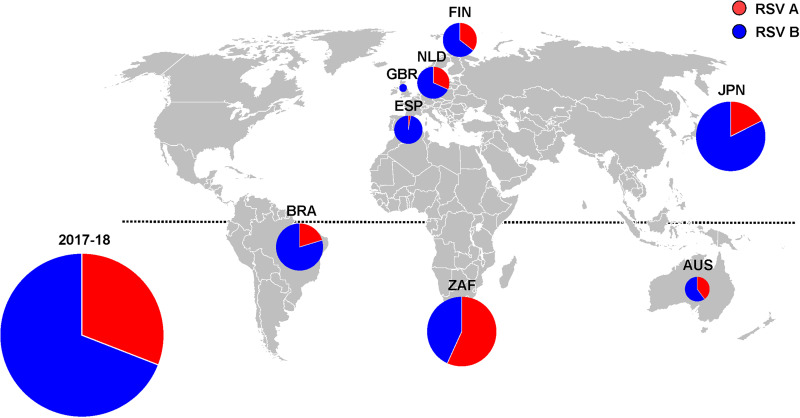
Geographic distribution of RSV A (*n* = 128) and RSV B (*n* = 283) subtypes, 2017–2018 (*n* = 8 countries). Overall size of the pies is proportional to the number of RSV isolates and the segments of the pies are proportional to the frequency of subtype A (red) and subtype B (blue) (Table 1). Northern hemisphere: GBR, United Kingdom (*n* = 2); ESP, Spain (*n* = 36); NLD, The Netherlands (*n* = 43); FIN, Finland (*n* = 45); JPN, Japan (*n* = 91). Southern hemisphere: BRA, Brazil (*n* = 64); ZAF, South Africa (*n* = 95); AUS, Australia (*n* = 34). (The figure was created with Microsoft PowerPoint.)

The INFORM-RSV study was initiated in 2017–2018 in 8 countries (United Kingdom [GBR], Spain [ESP], The Netherlands [NLD], Finland [FIN], Japan [JPN], Brazil [BRA], South Africa [ZAF], and Australia [AUS]) with an aim to expand to other countries where disease burden studies are ongoing (Fig. 1). RSV-positive nasal samples were collected in Universal Transport Medium from hospital-based laboratories as part of routine clinical care or specifically for research purposes and shipped to the University Medical Centre Utrecht for sequencing. Individual patient data collected included: location, sample date, age, gender, referring department, and length of hospital stay (18).

### RNA extraction, subtyping, RSV genome amplification, and next-generation sequencing.

Nucleic acids were extracted from RSV-positive nasal specimens using the MagNA Pure LC kit (Roche Diagnostics, Mannheim, Germany) as previously described (18). RSV subtyping and quantification were performed by multiplexed TaqMan RT-PCR analysis of the RSV N gene using RSV A and RSV B specific primer/probe mixes. Subsequently, subtype-specific RT-PCR was performed using the SuperScript IV one-step RT-PCR system (Invitrogen, Carlsbad, CA USA) to amplify 4 overlapping fragments covering the full RSV genome. The resultant 3.5 to 5.0 kb amplicons were pooled, purified from 1% agarose gels, used to construct libraries by means of the Nextera XT DNA Library Prep kit, and sequenced on a NextSeq 500 system (Illumina, San Diego, CA USA) (18).

### Sequence assembly and genotyping analysis.

Assembly of next-generation sequencing (NGS) reads into RSV G-F contigs was performed using AstraZeneca’s open-source NGS-Microbial Sequencing Toolbox, as previously described (18, 19). Alignment of RSV G HVR2 and full-length nucleotide sequences was performed in MUSCLE and evolutionary analyses of full-length RSV G sequences were conducted in MEGA7. Assignment of RSV genotypes was performed by phylogenetic clustering of RSV G HVR2 nucleotide sequences using a previously described 2014 reference database (11).

### Amino acid sequence variation analysis of RSV F proteins.

The RSV A and RSV B F sequences in FASTA format were translated into amino acid sequences and aligned against reference F sequences derived from year 2013 Netherlands RSV A/13-005275 (GenBank accession no: KX858757) and RSV B/13-001273 (GenBank accession no: KX858756) reference strains, respectively. Amino acid variation per position was determined and reported from pairwise alignments as previously described (18).

### Structural visualization of RSV F protein antigenic sites.

The 3D structures of prefusion and postfusion RSV F protein trimers were visualized with PyMOL molecular Graphics System, v2.2.2 (Schrödinger, LLC) using PDB 5UDE (12) and PDB 3RRR (20), respectively. Antigenic sites were defined using the six antibody epitopes (Ø and I to V) previously described (13).

### Statistical analyses.

A two-sided Fisher’s exact test was used to assess statistical significance of global subtype distribution among demographic categories and to compare proportions of amino acid changes between antigenic sites.

## RESULTS

### Geographic and demographic distribution of RSV A and B subtypes and genotypes.

**FIG 2:**
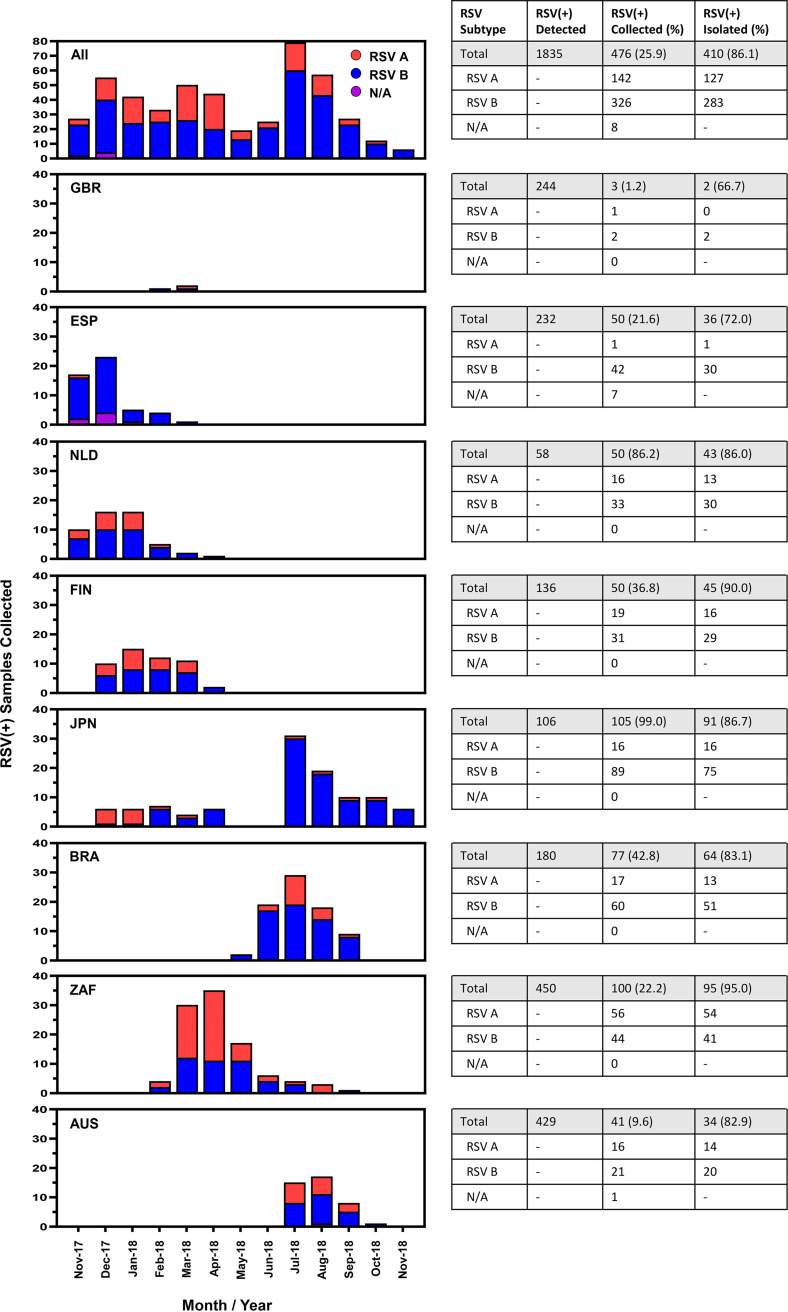
Monthly collection of RSV-positive(+) samples by country and overall number of RSV(+) detected, collected, and isolated/sequenced for RSV G-F gene analysis.

Between November 2017 and November 2018, 1,835 nasal samples tested RSV-positive among participating sites in 8 countries. Among the RSV-positive detections, 476 (25.9%) nasal samples were collected for inclusion in the INFORM-RSV study. The frequency and monthly pattern of RSV-positive samples collected from each country are shown in Fig. 2. Delayed study initiation resulted in fewer than the targeted 50 RSV-positive samples collected in 5 of the 8 countries. With some exceptions, the peak period for RSV-positive sample collection occurred from December to January and July to August in northern and southern hemisphere countries, respectively. Sequencing and assembly of full-length RSV G-F sequences was successful for 410 of the 476 (86.1%) RSV-positive samples, with even distribution between northern (52.9%; 217 of 410) and southern (47.1%; 193 of 410) hemispheres. The remaining 66 of 476 (13.9%) RSV-positive nasal samples failed sequencing due to unsuccessful RT-PCR amplification, insufficient sequencing depth, or low read quality. Among the 410 RSV strains with G-F sequence data, 127 (31.0%) were subtype A and 283 (69.0%) were subtype B. Overall, the proportion of RSV subtypes differed by country (*P < *0.001), as RSV B was more prevalent than RSV A in 7 of 8 countries studied, with the exception being South Africa (Fig. 1 and Table 1). Finally, genotype determination revealed that all RSV A strains were of the Ontario 1 (ON1) genotype and all RSV B strains were of the Buenos Aires 9 (BA9) genotype.

**TABLE 1:** Frequency of RSV A (*n* = 127) and RSV B (*n* = 283) subtypes by demographic and clinical characteristics and country

Country^*a*^	No. total (%)	Gender		Age			Length of Stay		Referring dept.^*b*^			
		No. male (%)	No. female (%)	No. <1 yr (%)	No. 1 to <2 yrs (%)	No. 2 to <5 yrs (%)	No. ≥24 h (%)	No. <24 h (%)	No. ER/ED (%)	No. PICU (%)	No. PW (%)	No. other (%)
All	410	231	179	333	59	18	289	121	25	39	74	272
RSV A	127 (31.0)	82 (35.5)	45 (25.1)	104 (31.2)	21 (35.6)	2 (11.1)	88 (30.4)	39 (32.2)	3 (12.0)	8 (20.5)	19 (25.7)	97 (35.7)
RSV B	283 (69.0)	149 (64.5)	134 (74.9)	229 (68.8)	38 (64.4)	16 (88.9)	201 (69.6)	82 (67.8)	22 (88.0)	31 (79.5)	55 (74.3)	175 (64.3)
GBR	2	0	2	2	0	0	2	0	0	1	1	0
RSV A	0	0	0	0	0	0	0	0	0	0	0	0
RSV B	2 (100.0)	0	2 (100.0)	2 (100.0)	0	0	2 (100.0)	0	0	1 (50.0)	1 (50.0)	0
ESP	36	20	16	31	3	2	31	5	5	0	0	31
RSV A	1 (2.8)	1 (5.0)	0	1 (3.2)	0	0	1 (3.2)	0	0	0	0	1 (3.2)
RSV B	35 (7.2)	19 (95.0)	16 (100.0)	30 (96.8)	3 (100.0)	2 (100.0)	30 (96.8)	5 (100.0)	5 (100.0)	0	0	30 (96.8)
NLD	43	23	20	40	1	2	39	4	1	22	19	1
RSV A	13 (30.2)	7 (30.4)	6 (30.0)	12 (30.0)	1 (100.0)	0	11 (28.2)	2 (50.0)	0	4 (18.2)	8 (42.1)	1 (100.0)
RSV B	30 (69.8)	16 (69.6)	14 (70.0)	28 (70.0)	0	2 (100.0)	28 (71.8)	2 (50.0)	1 (100.0)	18 (81.8)	11 (57.9)	0
FIN	45	27	18	45	0	0	4	41	0	0	0	45
RSV A	16 (35.6)	11 (40.7)	5 (27.8)	16 (35.6)	0	0	1 (25.0)	15 (36.6)	0	0	0	16 (35.6)
RSV B	29 (64.4)	16 (59.3)	13 (72.2)	29 (64.4)	0	0	3 (75.0)	26 (63.4)	0	0	0	29 (64.4)
JPN	91	43	48	46	33	12	43	48	5	0	54	32
RSV A	16 (17.6)	8 (18.6)	8 (16.7)	6 (13.0)	9 (27.3)	1 (8.3)	2 (4.7)	14 (29.2)	0	0	11 (20.4)	5 (15.6)
RSV B	75 (82.4)	35 (81.4)	40 (83.3)	40 (87.0)	24 (72.7)	11 (91.7)	41 (95.3)	34 (70.8)	5 (100.0)	0	43 (79.6)	27 (84.4)
BRA	64	42	22	59	5	0	44	20	14	13	0	37
RSV A	13 (20.3)	9 (21.4)	4 (18.2)	12 (20.3)	1 (20.0)	0	7 (15.9)	6 (30.0)	3 (21.4)	2 (15.4)	0	8 (21.6)
RSV B	51 (79.7)	33 (78.6)	18 (81.8)	47 (79.7)	4 (80.0)	0	37 (84.1)	14 (70.0)	11 (78.6)	11 (84.6)	0	29 (78.4)
ZAF	95	55	40	83	11	1	92	3	0	0	0	95
RSV A	54 (56.8)	35 (63.6)	19 (47.5)	47 (56.6)	6 (54.5)	1 (100.0)	52 (56.5)	2 (66.6)	0	0	0	54 (56.8)
RSV B	41 (43.2)	20 (36.4)	21 (52.5)	36 (43.4)	5 (45.5)	0	40 (43.5)	1 (33.3)	0	0	0	41 (43.2)
AUS	34	21	13	27	6	1	34	0	0	3	0	31
RSV A	14 (41.2)	11 (52.4)	3 (23.1)	10 (37.0)	4 (66.7)	0	14 (41.2)	0	0	2 (66.7)	0	12 (38.7)
RSV B	20 (58.8)	10 (47.6)	10 (76.9)	17 (63.0)	2 (33.3)	1 (100.0)	20 (58.8)	0	0	1 (33.3)	0	19 (61.3)

a GBR, United Kingdom; ESP, Spain; NLD, The Netherlands; FIN, Finland; JPN, Japan; BRA, Brazil; ZAF, South Africa; AUS, Australia.

b ER/ED, emergency room/department; PICU, pediatric intensive care unit; PW, pediatric ward; Other, other/undefined location.

Distribution of RSV strains by gender, age, and length of hospital stay was also determined. The median age of RSV-positive individuals was 5 months (interquartile range [IQR], 2 to 9 months) and 81.2% (333 of 410) were aged less than 1 year; 56.3% (231 of 410) were males; and 70.5% (289 of 410) were hospitalized for ≥24 h. RSV isolates from outpatients, characterized by a length of hospital stay of <24 h, were mostly derived from 3 countries (Finland, Japan, and Brazil) and accounted for 29.5% (121 of 410) of the total. Stratification by referring department revealed that most RSV isolates came from other/undefined locations (66.3%; 272 of 410), followed by the pediatric ward (PW) (18.0%; 74 of 410), emergency room/department (ER/ED) (6.1%; 25 of 410), and pediatric intensive care unit (PICU) (9.5%; 39 of 410) (Table 1). Overall, RSV B was more frequent than RSV A in all categories and there were no significant differences in the global proportion of subtypes by age group (*P = *0.141) or length of hospital stay (*P = *0.722). While a significantly higher proportion of RSV B cases were observed globally in females compared to males (*P = *0.0311), no gender differences were observed within individual countries.

### Global analysis of RSV genetic variability.

**FIG 3:**
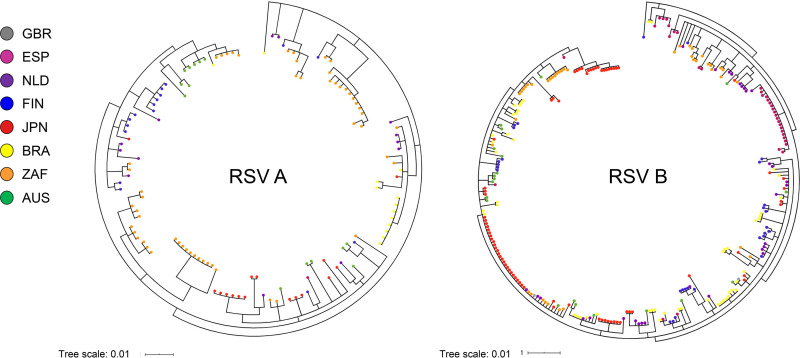
RSV A ON1 (*n* = 127) and RSV B BA9 (*n* = 283) G-based clades by country.

To understand genetic variability of the 2017−2018 RSV strains, we performed a phylogeographic analysis of G gene sequences by country. Within both RSV A (all ON1 genotype) and RSV B (all BA9 genotype) phylogenies, some sequences clustered within a country, suggesting microevolution, while other clusters contained sequences from multiple countries (Fig. 3). These data show that RSV A ON1 and RSV B BA9 strains from 2017–2018 were genetically diverse by geographic locale, consistent with wide transmission and continued evolution.

### Evidence for evolution of the RSV F protein.

**FIG 4:**
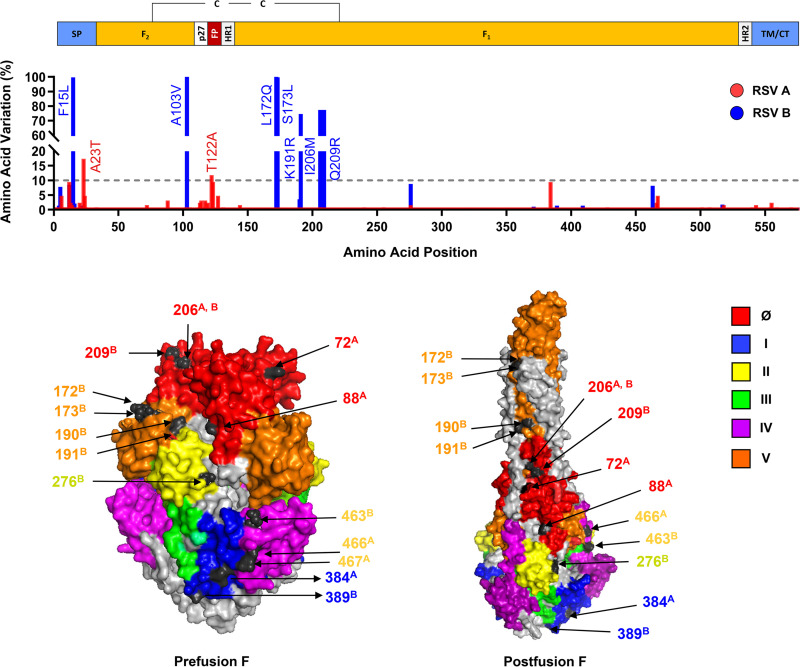
Individual frequency and structural location of amino acid polymorphisms in RSV A F (*n* = 127) and RSV B F (*n* = 283) protein sequences. (Top) Major structural features of full-length RSV F protein (amino acids [AA] 1–574), including the extracellular region (F_2_: AA 24–109 and F_1_: 137-524); SP, signal peptide; p27 peptide; FP, fusion peptide; HR, heptad repeats; and TM/CT, transmembrane/carboxy terminus. (Middle) Linear plot of individual amino acid variation frequency in full-length RSV A F (red) and RSV B F (blue) protein sequences compared to year 2013 RSV A/13-005275 and RSV B/13-001273 reference strains, respectively. Amino acid polymorphisms detected at ≥10% frequency (Table 2) are denoted. (Bottom) Proximal locations of amino acid polymorphisms in antigenic sites of mature prefusion and postfusion RSV F protein trimers. Previously defined antigenic sites (Ø and I to V) (13) are delineated in color. Amino acid positions at which polymorphisms were detected at ≥1% frequency (Table 2) are highlighted in black with adjoining arrows. A and B superscripts denote subtype A and B, respectively.

To assess recent evolution of the fusion protein, 2017–2018 RSV A F and B F protein sequences were compared to year 2013 RSV A/13-005275 and RSV B/13-001273 reference strains, respectively. Overall, diversity of RSV F sequences was low, with mostly conserved amino acid changes detected at 45 of 574 positions (7.8%) in RSV A F and at 62 of 574 positions (10.8%) in RSV B F (Fig. 4). Only 2 amino acid changes in RSV A F were highly polymorphic: A23T (17.3%) in the signal peptide and T122A (11.8%) in the fusion peptide. In contrast, 7 amino acid changes in RSV B F were detected in a majority of sequences as follows: F15L (99.6%) in the signal peptide, A103V (100%) in F_2_, and L172Q (100%), S173L (99.6%), K191R (74.2%), I206M (77.4%), and Q209R (76.3%) in F_1_.

Amino acid variation was further examined in each antigenic site (Ø and I to V) by geography (Table 2) and depicted on prefusion and postfusion F protein trimer structures (Fig. 4). No statistical differences in the global proportion of amino acid changes were observed between antigenic sites (data not shown) and some changes occurred in both RSV A F and B F at the same positions (Y33, I206, S255, and S276). Overall, 11 amino acid changes were detected in 4 of 6 antigenic sites for RSV A F, with frequencies ranging from 0.8 to 9.4%, and 32 amino acid changes were detected in 6 of 6 antigenic sites for RSV B F, with frequencies ranging from 0.4 to 100.0%. Only 5 of the 32 antigenic site changes in RSV B F were highly polymorphic and detected in all countries: I206M (77.0%) and Q209R (76.3%) in site Ø and L172Q (100.0%), S173L (99.6%), and K191R (74.2%) in site V. With few exceptions, antigenic site changes of intermediate polymorphic frequency (≥1% and <10%) were detected in multiple countries. These results indicate that F protein sequences and antigenic sites from 2017–2018 were generally well-conserved compared to year 2013 reference strains, although RSV B strains exhibited greater variability.

**TABLE 2:** Global frequency of amino acid polymorphisms in antigenic sites of RSV A F (*n* = 127) and RSV B F (*n* = 283) protein sequences and countries of detection

Site	Amino acid positions^*a*^	RSV A (*n* = 127)			RSV B (*n* = 283)		
		Change^*b*^	No. (%)	Country^*c*^	Change^*b*^	No. (%)	Country^*c*^
Ø	62–96; 195–227	T72A	2 (1.6)	ESP, NLD	K68R	1 (0.4)	AUS
		N88T	4 (3.1)	FIN	K68Q	1 (0.4)	JPN
		I206T	1 (0.8)	FIN	N201S	1 (0.4)	NLD
					**I206M**	**219 (77.4)**	**All**
					Q209K	1 (0.4)	NLD
					Q209L	2 (0.7)	BRA
					**Q209R**	**216 (76.3)**	**All**
I	27–45; 312–318; 378–389	Y33H	1 (0.8)	ZAF	Y33F	1 (0.4)	ZAF
		I384T	12 (9.4)	ZAF	P312H	1 (0.4)	NLD
					S380N	2 (0.7)	BRA
					L381I	1 (0.4)	FIN
					S389F	1 (0.4)	ZAF
					S389P	3 (1.1)	BRA
II	254–277	S255N	1 (0.8)	ZAF	S255G	1 (0.4)	ESP
		S276N	1 (0.8)	ZAF	M264I	1 (0.4)	FIN
		S276R	1 (0.8)	FIN	K272N	1 (0.4)	BRA
					L273I	1 (0.4)	ZAF
					S276N	25 (8.8)	BRA, ESP, FIN, GBR, NLD
III	46–54; 301–311; 345–352; 367–378				L303I	2 (0.7)	NLD
					I305T	1 (0.4)	ESP
					V349A	1 (0.4)	JPN
					N371S	3 (1.1)	JPN
IV	422–471	S425T	1 (0.8)	ZAF	K433R	1 (0.4)	AUS
		S466N	3 (2.4)	ZAF, NLD, JPN	L462Q	1 (0.4)	NLD
		L467I	6 (4.7)	ZAF, BRA	E463D	24 (8.5)	BRA, ESP, FIN, NLD
V	55–61; 146–194; 287–300				**L172Q**	**283 (100.0)**	**All**
					**S173L**	**282 (99.6)**	**All**
					K176R	1 (0.4)	AUS
					V179I	1 (0.4)	JPN
					S190N	10 (3.5)	BRA, ESP, FIN, JPN
					**K191R**	**210 (74.2)**	**All**
					V300I	1 (0.4)	FIN

a Amino acid positions that define antigenic sites Ø and I–V (13).

b Amino acid changes compared to year 2013 reference sequences; high-frequency polymorphisms (≥10%) are indicated in boldface type.

c GBR, United Kingdom; ESP, Spain; NLD, The Netherlands; FIN, Finland; JPN, Japan; BRA, Brazil; ZAF, South Africa; AUS, Australia.

## DISCUSSION

RSV A and B cocirculate during seasonal epidemic periods with alternating patterns of predominance over time (21). However, little is known about temporal evolution of RSV strains, global spread of unique genotypes, or how these factors relate to disease severity. Also important to the development of vaccines and MAbs is the need to identify and track patterns of F protein antigenic site changes, which may confer selective advantages in transmission or resistance. The INFORM-RSV study aims to describe global molecular evolution and epidemiology of RSV by prospectively monitoring temporal and geographical distribution of currently circulating strains. At the time of writing, the INFORM-RSV study has been ongoing for 3 years and is currently being conducted in 17 countries across 5 continents. The results herein provide baseline information on RSV strain distribution associated with different clinical parameters of disease severity and genetic variation of RSV G and F from 8 countries (GBR, ESP, NLD, FIN, JPN, BRA, ZAF, and AUS) across 4 continents during the 2017–2018 pilot season.

Genomic variation and evolutionary dynamics of RSV may affect its geographic, demographic, and clinical transmission behavior with important implications. During the INFORM-RSV 2017–2018 season, RSV B predominated over RSV A in all countries except South Africa, which may be attributed to virulence and local spread of RSV A strains specific to South Africa. Recent reports from North America (USA, 2015–2017 [22, 23]; Mexico, 2003–2015 [24]), Africa (ZAF, 2015–2017 [25]); [Kenya, 2000–2012 (26)], Asia (China, 2007–2015 [21]), and Australia (AUS, 2010–2016 [27]) describe alternating periodicity of RSV subtype prevalence, dominated by RSV A ON1 and RSV B BA9 genotypes. Consistent with these reports, RSV A ON1 and RSV B BA9 were the predominant genotypes of circulating RSV strains during the 2017–2018 RSV season. Geographic clustering patterns further suggest RSV transmission is characterized by continued genotype diversification during local spread and global dissemination.

Because the impact of viral factors on clinical parameters of disease severity has remained inconclusive (28), it was important to understand the distribution of RSV strains among demographic and clinical characteristics. Ultimately, most RSV strains were collected from hospitalized male infants aged less than 1 year, consistent with estimates of incidence and hospitalization rates (29), known risk factors, and the anatomic nature of shorter and narrower airways in infant males who are more likely to develop bronchial obstruction due to RSV infection (5). Unfortunately, the outpatient burden of RSV on health care resources has not been well defined (1, 2, 30) and few INFORM-RSV countries collected RSV-positive samples from outpatients who were medically managed without hospital admission. While hospital-based laboratory data on RSV infections may markedly underestimate the global burden of RSV disease, nevertheless, we observed no significant or meaningful differences in subtype/genotype distribution on clinical features of disease severity as assessed by gender, age group, or length of hospital stay.

The RSV F protein has historically been relatively well conserved, yet continues to evolve (12, 31). To that end, data from the INFORM-RSV 2017–2018 pilot season establishes an important molecular baseline of RSV F protein sequence and antigenic site variation from which to track frequency, geography, and evolutionary trajectory of potential neutralization escape variants as an early warning for vaccines and MAbs in development. Although the observed variability of the 2017–2018 RSV F sequences was low, with no differences in the proportion of amino acid changes between antigenic sites, the frequency and geographical distribution of some variants suggest a recent positive selection of favorable amino acid changes. Indeed, RSV B strains containing Q209R (site Ø) and L172Q/S173L (site V), first reported in China (2014–2016) (32), have recently emerged as dominant variants, with the addition of the I206M (site Ø) and K191R (site V) changes detected in the United States (2015–2019) (22, 33). These additional changes are possibly due to natural selective pressure from maternal or host neutralizing antibodies. Since site Ø and V elicit the greatest frequency of high-potency antibodies (34) in a structural area requiring a great deal of flexibility (13), these sites may tolerate greater amino acid variation than others. Additional, less frequent amino acid changes detected during the INFORM-RSV 2017–2018 study were frequent enough to be resampled in multiple countries but have yet to spread globally.

While the impact that widespread use of anti-RSV F MAbs will have on the emergence and transmission of resistant variants is unknown, these variants may also arise naturally in the absence of drug selection pressure. To date, palivizumab resistance-associated polymorphisms have been rarely observed in circulating RSV strains (35). Consistent with these reports, the restricted use of palivizumab (Synagis) (7), and the growth disadvantage of resistant variants in the absence of palivizumab selective pressure (36), we observed no known palivizumab target site II polymorphisms among 2017–2018 RSV strains. Also consistent with the rapid emergence and outgrowth of a RSV B strains containing L172Q/S173L in the United States (2015–2019) (22, 33), these nonconservative polymorphisms in suptavumab target site V were detected in 100% of global 2017–2018 RSV strains and coincide with clinical resistance and the recent failure of suptavumab to reduce overall RSV hospitalizations or outpatient LRTI in preterm infants in a phase 3 trial (6, 37). Finally, conservative I206M/Q209R polymorphisms in nirsevimab target site Ø were detected in 77% of RSV B strains but have been shown to retain susceptibility to neutralization by nirsevimab (38). Accordingly, despite the recent emergence of these polymorphisms, nirsevimab significantly reduced medically attended RSV LRTI in healthy preterm infants in a recent Phase 2b trial (9).

There are some limitations to the INFORM-RSV study. Key challenges to temporal analyses between geographies include adequate country representation and timing of RSV epidemics by season and location. Although low rates of RSV A and B coinfection (<2%) have been reported (22, 39), the use of subtype-specific primers/probes in the INFORM-RSV study did not permit detection of RSV A and B coinfection. Data on patients’ viral load are unavailable and therefore additional phylodynamic evolutionary and viral spread analyses are not possible. Since our data are heavily weighted toward infants with severe RSV disease that required hospitalization, we do not know about trends and molecular analyses of RSV from children who were medically managed as outpatients or were asymptomatic and did not seek medical attention. Our use of a 2014 RSV G HVR2 reference database (11) to genotype contemporary isolates has limitations as RSV continues to evolve. Accordingly, an extensible, centralized, curated, open database of reference sequences is needed to standardize genotyping and allow comparability across studies. Finally, future phenotypic susceptibility data would help to understand the functional impact of F protein antigenic site changes against anti-RSV F MAbs.

The strength of the INFORM-RSV study is reflected in its prospective design to characterize temporal and geographic trends in RSV diversity and to progress for several years with widespread global participation. Historically, RSV molecular epidemiology studies have been retrospective, focused exclusively on G gene diversity, and/or have been limited by geographical and low sampling effort constraints (15, 26, 40, 41). While global RSV surveillance is conducted by the European Influenza Surveillance Network (4) and the World Health Organization (42), none provide subtype differentiation or sequence analyses when reporting patterns of circulation. Findings from the INFORM-RSV study may have important implications in understanding the impact of RSV evolution on transmission, pathogenesis, and prophylaxis effectiveness. Tracking the frequency, recurrence, and distribution of amino acid changes that may confer selective advantages is a key focus of INFORM-RSV. Recent strains and dominant genotypes have genetic differences from the prototype virus strain used in most vaccine research (43). Since antigenic site changes could alter viral antigenicity for vaccines and affect their susceptibility to MAbs, novel agents for prophylaxis cannot afford to miss their contemporary targets when they are eventually deployed.

In conclusion, ongoing surveillance of global molecular epidemiology of RSV is important for detecting the emergence and spread of new strains, predicting their clinical impact, and providing an early warning system of antigenic changes that may affect the effectiveness of vaccines and MAbs. To that end, the INFORM-RSV 2017–2018 pilot season establishes an important molecular baseline of RSV strain distribution and sequence variability among hospitalized infants from which to investigate temporal and geographic relationships in the years ahead.
